# The Medicolegal Challenges of Facial Plastic Surgery: A Systematic Review

**DOI:** 10.1093/asj/sjaf082

**Published:** 2025-05-16

**Authors:** Saule A Mussabekova, Yuliya Menchisheva, Álvaro Varela Morillas

## Abstract

Facial plastic surgery is associated with a growing number of medicolegal challenges, particularly because of postoperative complications and patient dissatisfaction. This systematic review aimed to examine the medicolegal challenges associated with facial plastic surgery, focusing on postoperative complications, patient dissatisfaction, litigation cases, and medicolegal risk factors. A comprehensive literature search was conducted in MEDLINE (National Library of Medicine, Bethesda, MD), Embase (Elsevier, Amsterdam, the Netherlands), Cochrane Library (Wiley, Hoboken, NJ), PubMed (National Library of Medicine), Web of Science (Clarivate Analytics, Philadelphia, PA), SCOPUS (Elsevier), and Google Scholar (Alphabet Inc., Mountain View, CA) from 2020 to 2024. Medicolegal cases related to facial aesthetic surgeries were included. In total, 27 studies met the inclusion criteria. The leading causes of litigation included unsatisfactory aesthetic outcomes, failure of informed consent, technical surgical errors, and inadequate postoperative follow-up. The highest litigation rates were reported in countries with high volumes of plastic surgery cases and stringent regulations. This review highlights the increasing medicolegal burden in aesthetic surgery. Informed consent, postoperative monitoring, and technical precision are crucial for mitigating risks and preventing malpractice claims. The study synthesized medicolegal trends across all major facial aesthetic surgeries. It incorporated a global perspective, analyzing litigation data from over 10 countries, which is uncommon in most similar studies.

See the Commentary on this article here.

Aesthetic surgical procedures have experienced a surge in popularity globally, driven by increasing patient demand and advancements in surgical techniques.^1-4^ Advancements in surgical techniques, social and cultural shifts in beauty standards, and increased accessibility of aesthetic procedures drive the rise.^5-7^ Plastic surgery ranks among the top 5 specialties with the highest frequency of medicolegal cases.^2^ The physiological risks of plastic surgery are relatively lower than those in other surgical specialties.^8,9^ Aesthetic surgical procedures are typically elective and often performed on an outpatient basis in relatively healthy patients.^10^ Despite these factors, significant risks of postoperative complications exist.^11-13^ Common complications include infections, systemic toxicity of local anesthetics, electrolyte and hematological imbalances, intravascular fluid shifts, and wound complications.^5,9,12,14,15^ Consequently, dissatisfaction with surgical results, health deterioration, or severe harm can lead to medicolegal evaluations to determine the liability of physicians.^7,16-19^ Key questions in these evaluations often address cosmetic, medical, or functional defects; the alignment of surgical interventions with the treatment plan; and any harm caused to the patient's health, including the need for corrective surgery.^20-23^ Expert evaluations help determine whether the desired outcomes were achieved, establish if harm was caused to the patient's health, and assess the necessity and cost of corrective surgeries.^24-26^

An increased number of medicolegal claims underscores the importance of identifying the underlying factors contributing to litigation and implementing effective risk management strategies.^21,27,28^ Several studies focusing on facial plastic procedures, such as rhinoplasty, blepharoplasty, and facelifting, have noted the critical role of informed consent in mitigating legal risks.^4,7,29-32^ Studies have consistently shown that claims involving inadequate or missing informed consent often result in legal judgments against the surgeon, regardless of surgical competence.^20,33,34^

The practice of facial plastic surgery is particularly susceptible to legal scrutiny because of its highly subjective outcomes.^8,12^ Unlike reconstructive procedures, where the goal is to restore form and function, aesthetic procedures are often judged against personal expectations and idealized results.^22,27^ As a result, even technically successful surgeries can result in claims if the patient's desired appearance is not achieved.^35,36^ Understanding the complex interplay between surgical techniques, patient expectations, and legal considerations is crucial for plastic surgeons.^10,37^ Several studies emphasize the need for improved communication and realistic expectations to avoid possible dissatisfaction postsurgery.^6,30,38^ Analysis of malpractice claims data also suggests an increasing litigation rate with aesthetic surgeries, making it more important to improve methods to reduce legal risks and patient communication.^7,33^

Another contributing factor to litigation is the variability in legal frameworks and healthcare regulations across different countries.^39,40^ High-income countries with stringent regulatory systems and a litigious culture tend to report higher volumes of legal cases in aesthetic medicine.^37,41^ In contrast, lower income countries may experience underreporting because of limited access to legal resources or less transparency in healthcare litigation.^21,42^ Consequently, the medicolegal environment is not uniform and can significantly influence both the incidence and outcomes of malpractice claims.

Numerous studies have attempted to analyze the medicolegal landscape of facial plastic surgery, often drawing data from national malpractice databases, legal registers, and forensic case reviews.^5,42^ These analyses have provided valuable insights into the nature of claims, types of procedures most commonly implicated, and the legal arguments employed by plaintiffs and defense attorneys.^43,44^ However, a consolidated synthesis of such data has been lacking, making it difficult to develop evidence-based guidelines for risk reduction.

This systematic review aimed to comprehensively examine the medicolegal challenges associated with facial plastic surgery and identify common complications leading to medicolegal cases for reducing malpractice claims.

## METHODS

The protocol for this study was registered in PROSPERO (ID: CRD420251002669; https://www.crd.york.ac.uk/PROSPERO/view/CRD420251002669) and compiled by the Preferred Items for Systematic Reviews and Meta-Analyses (PRISMA) reporting guidelines.^45^ The study selection process is illustrated using the PRISMA flowchart.^46^ The Appendix has more details on the search methodology.

### Eligibility Criteria

Specific inclusion and exclusion criteria to ensure the selection of relevant studies were established (Supplemental Table 1). The inclusion criteria encompassed studies involving adults aged 18 and above of any gender who underwent facial plastic surgical procedures. These procedures included brow lifts, upper and lower lid blepharoplasty, facelifts (rhytidectomy), neck lifts (platysmaplasty), rhinoplasty, volume augmentation, genioplasty, malarplasty, lip lifts, dimple creation, and buccal fat removal. The selection focused on studies reporting complications following these surgeries, as well as cases of patient dissatisfaction categorized under medical malpractice, professional misconduct, litigation for medical negligence, license revocation, claims for moral damages, forensic medical disputes, compensation claims for medical harm, and adverse outcome liability. Only articles published in English from 2020 onwards were analyzed, specifically prospective and retrospective studies, case reports, case series, cohort studies, and descriptive studies.

Conversely, the exclusion criteria ruled out studies involving adults who underwent plastic surgery in anatomical regions other than the face, as well as those focusing on nonsurgical aesthetic procedures, such as injectable treatments with hyaluronic acid fillers, botulinum toxin, hydroxyapatite injections, laser ablations, and tattooing. Studies reporting complications arising from dental practices or reconstructive surgeries in the maxillofacial region following trauma or addressing congenital and acquired defects and deformities were also excluded. Non-English articles, those published before 2020, and publications in the form of commentaries, editorials, letters, reviews (systematic or otherwise), guidelines, position papers, in vitro, and animal studies were excluded.

### Information Sources

A comprehensive literature search was conducted across the following 7 electronic databases (MEDLINE, Embase, Cochrane Library, PubMed, Web of Science, SCOPUS, and Google Scholar); 2 registers (ClinicalTrials.gov and the World Health Organization International Clinical Trials Registry Platform [WHO ICTRP]); and 1 website: WorldCat. The date range for all searches was 2020 to 2024. Citation searching yielded an additional 12 records. Gray literature sources were also searched from platforms including ProQuest Dissertations and Theses (PQDT; Clarivate), Open Access Theses and Dissertations, and the King's College London Research Portal. Conference proceedings were searched through SCOPUS and Web of Science, whereas preprints were checked from SSRN and F1000 Preprints. Supplemental Tables 2 and 3 provide detailed information pertaining to the supplemental search strategies. The last update on searches occurred on March 31, 2025.

### Search Strategy

A detailed literature search strategy was developed in conjunction with an experienced research librarian to guarantee the thorough inclusion of all relevant studies, regardless of language, study type, or publication status, encompassing published, unpublished, in-press, and ongoing research.

The Population, Intervention, Comparison, Outcome (PICO) framework was applied to ensure a structured and systematic approach to formulate the research question and guide the literature search (Supplemental Table 4). Research question: in patients dissatisfied with facial plastic surgery outcomes (P), how do medicolegal evaluations (I) impact risk management in medical practice (O)?

PICO framework:

- Population (P): individuals who underwent facial aesthetic surgical procedures.
- Intervention (I): evaluation of postoperative complications and medicolegal disputes.
- Comparison (C): not applicable.
- Outcome (O): identification of causes of medicolegal challenges, common complications, and proposed solutions.

The following combinations of medical subject headings, entry terms, and key words were used in the electronic search: “human,” “face,” “face surgery,” “facelift,” “plastic surgery,” “plastic surgery procedures,” “cosmetic surgery,” “aesthetic surgery,” “aesthetics,” “liability,” “legal,” “malpractice,” “legislation,” “forensic medicine,” “medical errors,” “compensation,” and “redress” (Supplemental Table 5). Search strategy was adapted to each database presented in Supplemental Tables 2 and 3. The PRESS Checklist was used to assess the search tactics.^47^

### Selection Process

Automated screening tool—Rayyan AI (Qatar Computing Research Institute) was used for selection. All retrieved studies underwent a 2-phase screening process independently by 2 reviewers (S.A.M. and Y.M.) to determine eligibility at each stage of the selection process.

### Data Collection Process

EndNote, a reference management software, was utilized to streamline the data collection and organization process. Duplicate articles were removed. The articles were selected by title and abstract for relevance according to the specified inclusion and exclusion criteria outlined below. All abstracts were independently reviewed. Any disagreements were resolved by the third author's involvement (A.V.M.).^48^ Google Scholar Alerts was used to update citations extended to March 31, 2025 in order to ensure the inclusion of the most recent and relevant publications.

Articles that satisfied the requirements for full-text review were extracted. A standardized data extraction form was used to collect information on study characteristics (author, year, country, and study type), total cases analyzed and types of facial plastic surgery procedures involved, primary medicolegal allegations and legal outcomes, common postoperative complications, and risk factors for litigation.

### Study Risk of Bias Assessment

The risk of bias assessment was conducted using 2 validated tools based on the study design. The Joanna Briggs Institute (JBI) critical appraisal checklist (JBI, Australia) was used for case reports and case series.^49^ For retrospective and prospective studies, the Risk of Bias in Non-Randomized Studies of Interventions (ROBINS-I; Cochrane, United Kingdom) was applied to assess potential biases in studies.^50^ Two independent reviewers (S.A.M. and Y.M.) conducted a risk of bias assessment. Supplemental Tables 6-8 provide detailed documentation of the assessment process.

## RESULTS

**Figure 1.**
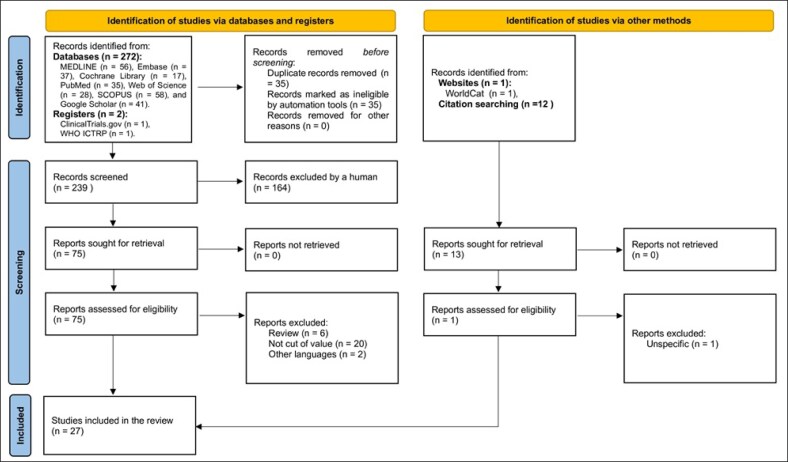
Preferred Items for Systematic Reviews and Meta-Analyses flowchart of the selection process.

A total of 27 studies were included in this systematic review (Supplemental Table 9). Excluded studies are presented in Supplemental Table 10. The PRISMA flow diagram (Figure 1) illustrates the systematic selection process for study inclusion. Selected studies covered medicolegal cases related to facial plastic surgery across multiple countries, including the United States of America, Spain, Brazil, Canada, the United Kingdom, Japan, Italy, Turkey, Iran, and Belgium. Out of 27 studies, 13 were classified as retrospective (48.1%), which included 12 observational studies (44.4%). Additionally, 4 studies were identified as cross-sectional (14.8%), and 4 were designated as case studies (14.8%), encompassing 1 case series. The studies utilized legal databases to extract malpractice claims and their outcomes, thereby underscoring a significant emphasis on retrospective analyses within the domain of plastic surgery malpractice. The qualitative characteristics show a diverse range of study designs aimed at understanding clinical outcomes, patterns, and associations in medical practice. The timeframes of cases analyzed varied significantly across studies, with some spanning multiple decades. For instance, the study by ElHawary et al examined cases from 1970 to 2020, representing a comprehensive exploration of over 50 years of malpractice litigation in plastic surgery.^51^ Conversely, Zhang et al, focused on more recent periods, analyzing cases from 2013 to 2017, whereas Feola et al reviewed cases from 2012 to 2016.^52,53^ Additionally, Navaratnam et al analyzed clinical negligence claims from 2013 to 2018, specifically focusing on rhinology and facial plastic surgery in England.^54^

### Study Characteristics

**Figure 2.**
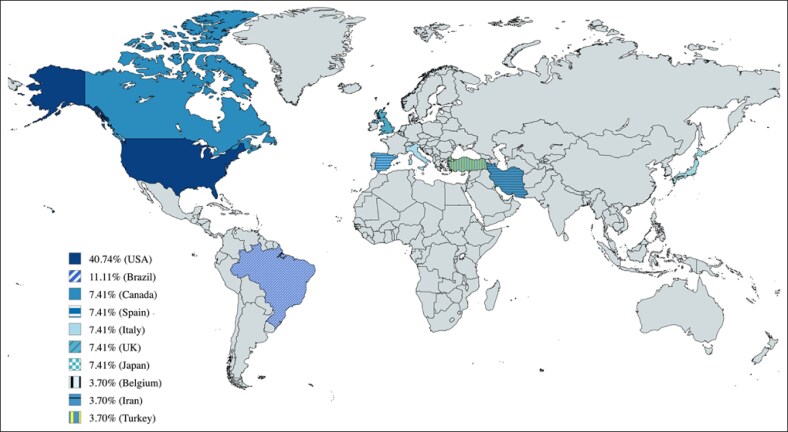
Study the distribution by nation.

The total number of legal cases analyzed across all studies ranged from 105 to 750 cases per study, with significant variations based on geographic location and database access. The studies were conducted across multiple countries, with the following distribution: the United States of America (11 studies), Canada (2 studies), Spain (2 studies), Italy, (2 studies), Japan (2 studies), Brazil (3 studies), Turkey (1 study), the United Kingdom (2 study), Belgium (1 study), and Iran (1 study).^51-76^ This geographical diversity underscores the global relevance of plastic surgery litigation (Figure 2).

From 2020 to 2024, studies were conducted with the following distribution: 2020 (3 studies [11.11%]), 2021 (9 studies [33.33%]), 2023 (10 studies [37.04%]), and 2024 (5 studies [18.52%]). The year 2023 marked a peak in research activity, primarily driven by contributions from the United States of America, with significant representation in journals dedicated to aesthetic and reconstructive surgery. The primary focus of litigation in facial plastic surgery involved aesthetic procedures, including rhinoplasty, facelifts, blepharoplasty, and facial implants. Some studies also included reconstructive procedures, although these were less frequently litigated.

The studies were clustered into 3 main categories based on their focus. Table 1 summarizes a list of studies describing an overview of the frequency and nature of malpractice claims across different surgical specialties, which are categorized as “General trends in malpractice.”^46-48,50,52,59,62,64,66^ The publications outlined in Table 2 grouped under “Procedure-specific” analysis and focused on particular surgeries such as rhinoplasty and blepharoplasty, examined the unique challenges, complications, and litigation patterns associated with these procedures.^49,51,53,56,57,60,63,65,71,72^ The investigations featured in Table 3 are classified under “Legal and ethical considerations.”^54,55,58,61,67-69^ These studies delved into the implications of informed consent, the role of expert witnesses, and the legal frameworks surrounding malpractice claims.

**Table 1.** The Studies Clustered into General Trends in the Malpractice Group

Authors, year, country	Study type; sample size; databases; timeframe	Complications	Common allegations	Legal outcomes
ElHawary et al^51^, 2023, Canada	Observational, retrospective study; 105 cases; LexisNexis, Westlaw Next; 1970-2020	Cosmetic deformity, poor scarring	Inadequate clinical judgment, lack of informed consent	64.2% in favor of plastic surgeons; avg. damages CAN$61,076
Zhang et al^52^, 2021, Canada	Observational, retrospective study; 414 cases; The Canadian Medical Protective Association; 2013-2017	Cosmetic deformity	Deficient clinical assessment, inadequate informed consent	47.8% unfavorable for plastic surgeons; 43.4% of patients compensated
Feola et al^53^, 2021, Italy	Retrospective study; 144 judgments; observatory project on medical responsibility; 2012-2016	Scarring, general dissatisfaction with outcomes, and postoperative complications	Inadequate or no informed consent (34% of judgments), dissatisfaction with outcomes (25.6%)	70.14% of claims accepted; professionals found liable in 63.89% of cases; total compensation €4,727,579
Sarmiento et al^55^, 2020, USA	Retrospective study; 165 cases; Westlaw; 2000-2017	Disfigurement, injury, psychological distress; most errors occurred intraoperatively	Gross negligence, informed consent deficiencies, surgical complications, and follow-up issues	60% ruled in favor of surgeons; verdicts primarily by judges (73%); settlements in 2% of cases
Boyd et al^57^, 2021, USA	Retrospective study; 3008 claims; Medical Professional Liability Association, Applied Medico-Legal Solutions; 1980-2020	Poor aesthetic outcomes	Procedural errors, lack of informed consent	75% of claims result in no payment
Remington et al^64^, 2024, USA	Retrospective study; 2674 cases; Candello; 2009-2018	Emotional trauma, surgery-related complications	Poor surgical technique, communication issues	26.8% resulted in indemnity payments
Martin-Fumado et al^67^, 2023, Spain	Retrospective study; 1039 claims; The Council of Medical Associations of Catalonia; 1986-2018	Allegations related to poor aesthetic results, informed consent issues	Lack of information, poor cosmetic outcomes	Liability was observed in 21.46% of closed claims; average compensation amounts varied by claim type
Taniguchi et al^69^, 2023, Japan	Descriptive study; 13,340 claims; Supreme Court data; 2006-2021	Nonfatal injuries	Negligence, lack of informed consent	75.8% ruled in favor of medical professionals
Mariani et al^71^, 2020, Brazil	Retrospective, cross-sectional study; 360 cases; CREMESP; 2007-2016	Surgical errors, inadequate treatment methods, improper postoperative care	Malpractice, medical advertising, poor doctor–patient relationships	19.9% of doctors judged found guilty of negligence or professional malpractice; 2.23% of cases dismissed

**Table 2.** The Studies Clustered into Procedure-Specific Analysis

Authors, year, country	Study type; sample size; databases; timeframe	Complications	Common allegations	Legal outcomes
Navaratnam et al^54^, 2022, England	Retrospective study; 171 claims; NHS Resolutione; 2013-2018	Intraoperative complications, unnecessary operations, and visual disturbances	Unnecessary pain, unnecessary operation, consent issues	119 closed claims with damages paid in 55 cases; total estimated costs of £13.6 million
Gibstein et al^56^, 2023, USA	Retrospective study; 21 cases; LexisNexis; 1988-2020	Procedural-related adverse outcomes, including scarring, infections, and hypoxic brain injury	Lack of informed consent, procedural error, failure to supervise trainees, inexperience of trainee	Verdicts in favor of the defense in 38.1% of cases; median payout for plaintiff-won cases was $5,100,000
Ong et al^58^, 2021, USA	Retrospective study; 23 cases; Westlaw; 1960-2018	Poor aesthetic outcomes, disfigurement	Technical errors, inadequate informed consent	86.9% ruled in favor of surgeons
Brozynski et al^61^, 2024, USA	Retrospective study; 46 cases; Westlaw, LexisNexis; 1987-2018	Burn injuries, including postoperative burns and complications arising from mishandled instruments	Mishandling of cautery devices, overheated instruments, human error, and neglect	43% of cases ruled in favor of defendants; 33% favored plaintiffs; 15% settled; no cases dismissed
Ceremsak et al^62^, 2021, USA	Retrospective study; 94 cases; LexisNexis, Westlaw; 2010-2019	Penetration of adjacent structures, hemorrhage, laryngeal nerve injury, cerebrovascular accidents	Improper surgical performance (49%), failure to diagnose (32%), lack of informed consent	89% of cases ruled in favor of defendants; average indemnity for plaintiff-won cases was $4.24 million
Venditto et al^65^, 2024, USA	Case series; 10 cases; clinical questionnaires; 2020-2023	Hemifacial paralysis	Not explicitly mentioned	Not detailed; focus on clinical outcomes
Defraia et al^68^, 2024, Italy	Case study; 1 case; clinical questionnaires; 2023	Lagophthalmos, missed hyperthyroidism	Medical malpractice because of alleged over-resection during blepharoplasty	Lagophthalmos attributed to underlying thyroid disorder; compensation not due
Nagano et al^70^, 2024, Japan	Case study; 1 case; clinical questionnaires; 2024	Cervical hematoma, upper airway obstruction, cardiopulmonary arrest	Inadequate monitoring, informed consent issues, and technical errors	No charges were filed against the surgeon; legal implications noted for complications
Ross et al^75^, 2023, UK	Retrospective study; 31 claims; National Health Service (NHS) Resolution; 2015-2020	Intraoperative complications, inadequate postoperative care, and misclassification of dissatisfaction	Intraoperative problems (32%), failure to warn/informed consent (19%), and foreign body left in situ	Total cost of claims was £1,347,336; significant financial implications noted; claims related to informed consent totaled £261,625.90
Ghorbani et al^76^, 2023, Iran	Retrospective, cross-sectional study; 117 cases; forensic medicine archive; 2011-2020	Respiratory problems	Lack of informed consent	Not specified; dissatisfaction can lead to legal action
Dhooghe et al^77^, 2022, Belgium	Case study; 50 cases; clinical questionnaires; 2012-2022	Fat embolism	Procedural complications	7 fatalities; significant financial damages reported

**Table 3.** The Studies Clustered into Legal and Ethical Considerations

Authors, year, country	Study type; sample size; Databases; timeframe	Complications	Common allegations	Legal outcomes
Ziai et al^59^, 2021, USA	Retrospective study; 186 cases; Westlaw, LexisNexis; 1919-2020	Facial nerve damage	Improper performance, failure of informed consent	58.1% ruled in favor of defendants
Halepas et al^60^, 2021, USA	Retrospective study; 55 cases; Westlaw; 2010-2020	Scarring, permanent injury	Informed consent issues	74% in favor of defendants
Moura et al^63^, 2023, USA	Retrospective study; 64 cases; Westlaw; 1979-2022	Permanent injury, disfigurement	Informed consent issues	60.9% in favor of physicians; median payout $340,520
Vicente-Ruiz et al^66^, 2025, Spain	Observational, retrospective study; 199 court resolutions; CENDOJ, ARANZADI, VLEX; 2018-2022	Aesthetic dissatisfaction	Unsatisfactory surgical results, technical errors	50% of cases were ruled in favor of the defendants
Grillo et al^72^, 2023, Brazil	Retrospective, cross-sectional study; 122 judgments; Justice Court of the São Paulo State 2012-2022	Postoperative bleeding, aesthetic dissatisfaction, and potential medical errors	Inadequate informed consent, dissatisfaction with aesthetic outcomes, medical errors during procedures	63 cases favored the defendant, 58 favored the plaintiff; average financial damages were $59,536
de Menezes et al^73^, 2020, Brazil	Retrospective study; 90 cases; Justice Department of Minas Gerais; 2000-2015	Unsightly scars, asymmetries, necrosis, infection	Unspecified dissatisfaction, inadequate informed consent, technical errors	Mixed outcomes; surgeons were often favored when expert reports supported their conduct
Yücel et al^74^, 2024, Turkey	Retrospective study; 100 cases; court records and institutional files; 2011-2022	Insufficient or lack of informed consent, inadequate clinical judgment, diagnostic errors	Insufficient or lack of informed consent (54.2%), inadequate clinical judgment, treatment errors	88.7% of patient-favored lawsuits were because of negligence

### Risk of Bias in Studies

The appropriate checklist was selected based on the research design: the JBI Critical Appraisal Checklist for case reports was applied to 4 studies, whereas the ROBINS-I Checklist for observational cohort and cross-sectional studies was utilized for the remaining 23 studies.^65,68,70,77,78^ Each study was meticulously reviewed against the specified criteria. A summary of these assessments is provided in Supplemental Tables 8-10. An overarching appraisal was derived: case reports led to straightforward inclusion or exclusion decisions, whereas observational studies were rated for bias as “low,” “moderate,” “high,” or “unclear” based on the criteria met. The overall risk-of-bias assessments indicate that the majority of studies exhibit a moderate risk across various domains, thereby contributing to the internal validity of the systematic review findings.

### Common Allegations and Legal Outcomes

Across studies, common allegations against surgeons included: intraoperative complications (nerve damage, infection, and scarring), poor aesthetic outcomes (dissatisfaction with cosmetic appearance), lack of informed consent, and failure to diagnose or manage complications appropriately (Table 4). A recurrent theme across studies was the significance of informed consent in mitigating malpractice claims.^51,53,56,58,68,72^ Issues surrounding inadequate consent processes were frequently cited as contributing factors to litigation. Many studies assessed patient-reported outcomes, focusing on dissatisfaction postsurgery.^56,58,63,64,72,75,76^ The link between aesthetic outcomes and litigation was highlighted, particularly in aesthetic procedures where patient expectations played a crucial role. An upward trend in litigation related to facial plastic surgeries has been observed. The studies indicated that the frequency of claims is increasing, particularly in aesthetic surgeries, which were more prone to patient dissatisfaction.^58,76^ The involvement of various surgical specialties in malpractice cases was significant. The data indicate that plastic surgeons, otolaryngologists, and maxillofacial surgeons frequently faced litigation, often reflecting the complexities of the procedures performed.

**Table 4.** The Reasons Contributing to Medicolegal Problems

№	Reason	Explanation	Studies
1	Out-of-scope practice	Nonplastic surgeons performing cosmetic procedures	Moura et al^63^
2	Improper performance/surgical errors	Surgical mistakes leading to complications or dissatisfaction	ElHawary et al^51^, Zhang et al^52^ , Vicente-Ruiz et al^66^, Sarmiento et al^55^, Martin-Fumado et al^67^, Gibstein et al^56^, Boyd et al^57^, Feola et al^53^, Navaratnam et al^54^, Ong et al^58^, Taniguchi et al^69^, Ziai et al^59^, Halepas et al^60^, Brozynski et al^61^ , Ceremsak et al^62^, Ross et al^62^ , Grillo et al^72^, Nagano et al^70^ , Dhooghe et al^77^, Remington et al^64^
3	Lack of informed consent or inadequate informed consent	Failure to properly educate patients on risks and alternatives	ElHawary et al^51^, Zhang et al^52^,Vicente-Ruiz et al^66^, Sarmiento et al^55^,Martin-Fumado et al^67^, Gibstein et al^56^, Boyd et al^57^, Feola et al^53^, Moura et al^63^, Navaratnam et al^54^, Ong et al^58^, Taniguchi et al^69^, Ziai et al^59^, Halepas et al^60^, Ghorbani et al^76^, Ross et al^75^, Grillo et al^72^, Nagano et al^70^
4	Failure to diagnose, refer, or treat	Misdiagnosis or delayed diagnosis, inadequate monitoring	ElHawary et al^51^, Zhang et al^52^, Boyd et al^57^, Navaratnam et al^54^, Taniguchi et al^69^, Ceremsak et al^62^, Nagano et al^70^
5	Aesthetic dissatisfaction	Unrealistic expectations or poor outcomes in cosmetic surgery	Vicente-Ruiz et al^66^, Martin-Fumado et al^67^, Feola et al^53^, Ong et al^58^, Halepas et al^60^, Ghorbani et al^76^, Grillo et al^72^
6	Communication failures	Poor doctor–patient communication leading to misunderstandings and unmet expectations	Sarmiento et al^55^, Navaratnam et al^54^ , Remington et al^64^
7	Failure to supervise a trainee	Lack of oversight in resident cases	Gibstein et al^56^

Expert witnesses played a critical role in court proceedings, with their involvement documented in 62% to 85% of cases.^52,63,66,73,75^ Their opinion often influenced case outcomes, particularly in establishing whether standard care protocols were followed.

Resident involvement was rarely documented, but in studies where it was addressed, residents were involved in <5% of cases, with no significant difference in litigation outcomes compared with attending surgeons.^55,56,60^

Reported complications were diverse and procedure specific: permanent injury, nerve injuries, burns, respiratory problems/nasal asymmetry, visual disturbances/dry eye, disfigurement, infections, scarring, fat embolism, penetration of adjacent structures, bleeding, and hematomas.^52,58,59,70^

The legal outcomes varied. Dismissal of cases ranged from 45% to 76% across different studies. Settlements observed in 20% to 40% of cases, and higher damages paid noted in cases involving severe functional impairment rather than dissatisfaction alone.

Although a substantial number of cases resulted in verdicts favoring the plaintiff, this may reflect a selection bias inherent in the datasets used, as only cases proceeding to litigation are included. Weaker cases are often dismissed prelitigation or settled confidentially and thus may not be captured in legal databases. This limitation further underscores the need for more comprehensive medicolegal data collection.

### Recommended Measures for Surgeons to Mitigate Medicolegal Risks in Facial Aesthetic Surgery

To effectively mitigate medicolegal risks in facial aesthetic surgery, surgeons should prioritize thorough preoperative counseling to align patient expectations with realistic outcomes. Detailed and well-documented informed consent discussions are essential, including disclosure of risks, alternatives, and limitations of the procedure. Psychological screening, particularly for conditions like body dysmorphic disorder, can help identify high-risk patients prone to dissatisfaction. Ongoing training in medicolegal education and proper postoperative follow-up further enhance patient safety and reduce litigation potential. A comprehensive list of these evidence-based strategies is presented in Table 5.

**Table 5.** Measures to Mitigate Medicolegal Risks in Facial Aesthetic Surgery

Preventive measure	Description
Enhanced preoperative counseling	Conduct detailed discussions about risks, benefits, and realistic expectations before surgery
Comprehensive documentation	Maintain thorough records of consent forms, operative plans, intraoperative notes, and discharge instructions
Objective outcome measures	Use validated tools (eg, aesthetic assessment scales) to measure and communicate surgical outcomes
Improved patient selection	Select patients carefully based on medical and psychosocial suitability for elective aesthetic procedures
Psychological screening	Implement tools to identify patients with unrealistic expectations or body dysmorphic disorder
Standardized informed consent protocols	Use digital aids and checklists to ensure patients are fully informed before signing consent
Resident supervision and training	Ensure appropriate oversight and documentation in cases involving trainees or junior surgeons
Legal and ethical education	Include medicolegal training modules in residency programs to build awareness of litigation risks
Postoperative follow-up	Schedule structured follow-up visits to monitor recovery and manage complications early
Use of expert witness support	Consult and involve expert witnesses early in the legal process to strengthen case defense

### Statistical Findings

**Figure 3.**
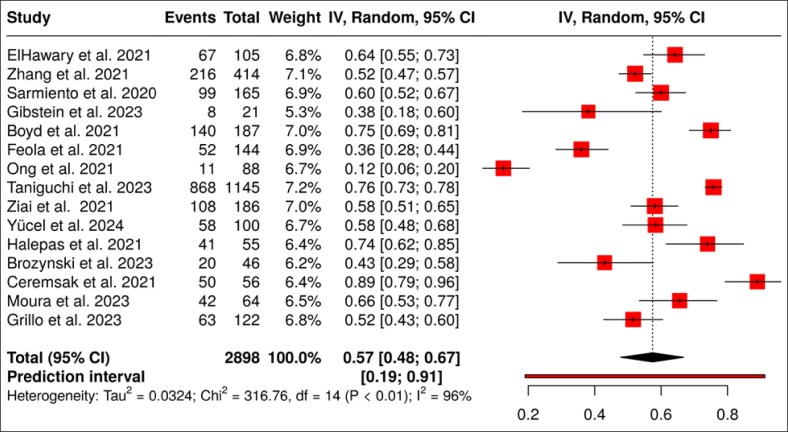
Forest plot of legal outcomes decided in favor of surgeons in plastic surgery.

This analysis aimed to synthesize available data on malpractice litigation related to plastic surgery, focusing on legal outcomes and the frequency of verdicts in favor of the operating surgeon. Out of 27 studies, only 15 provided complete information on the total number of litigated cases, the number and percentage of cases resolved in favor of the surgeon, and statistical evaluation. This selection allowed for calculation of proportions of favorable outcomes and the construction of a forest plot with 95% CIs, enabling a robust descriptive comparison of litigation patterns in facial plastic surgery internationally (Figure 3).

Most studies clustered around the 50% to 60% range. A few studies fell significantly below this average, likely because of patient selection bias or region-specific legal pressures. The overall summary proportion hovered at ∼54.3% (95% CI, 49%-59%).

Although not all studies performed statistical analysis, those that did identify significant correlations: higher litigation risk in elective cosmetic procedures (compared with reconstructive surgery), more frequent patient victories when improper consent procedures were documented, and no significant association between resident involvement and higher litigation risk.

### Limitations Noted in Studies

Several studies acknowledged limitations, including geographical restrictions (cases limited to national databases), incomplete case records (missing details on settlements), and potential underreporting of malpractice claims in certain jurisdictions.^51-54,59,66,67,69,71,77^ Legal research databases (eg, Westlaw [Thomson Reuters, Toronto, ON, Canada], LexisNexis [RELX plc, London, United Kingdom]) had intrinsic limitations.^55,62,63,70^ They primarily include cases that proceed to court and exclude a significant portion of prelitigation resolutions, such as confidential settlements, arbitration outcomes, or informal dispute resolutions. These precourt proceedings are neither documented in these databases nor subject to analysis, which limits the full understanding of medicolegal challenges in facial plastic surgery.^52,53,55,69,77^ Furthermore, data reporting requirements varied by jurisdiction, which affected the comprehensiveness of the available information.^52,63,69,71^

**Figure 4.**
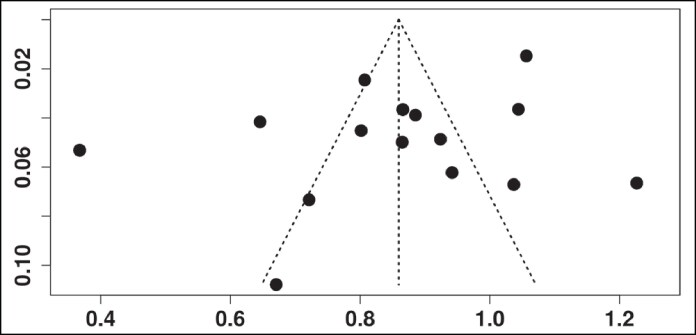
Funnel plot assessing publication bias in studies reporting legal outcomes in facial plastic surgery.

A formal assessment of publication bias was performed using a funnel plot based on the proportion of verdicts in favor of surgeons from 15 studies that reported complete legal outcomes. The plot examined the relationship between the effect size (success rate in litigation) and standard error across studies. Visual inspection of the funnel plot revealed a generally symmetrical distribution around the mean proportion (∼54.3%), indicating no strong evidence of publication bias (Figure 4). However, mild asymmetry was observed at the extremes, particularly among studies with smaller sample sizes, which may reflect underlying heterogeneity in study designs, regional legal systems, or litigation reporting practices rather than systematic bias. Although a statistical test such as Egger's regression could offer further insights, its utility is limited in the context of diverse study methodologies and a relatively small number of large-scale comparable datasets. Nevertheless, the inclusion of studies from multiple countries and legal systems helped reduce the risk of selective reporting and enhanced the robustness of this review's findings.

## DISCUSSION

Initial screening revealed that many studies reporting on litigation in facial plastic surgery were heterogeneous in both scope and format. Several studies included case reports or focused only on thematic aspects, whereas others lacked clear numerical outcome data.^59,61,62,65,68,70,75-77^ Additionally, methodologies varied substantially. Some relied on insurance claim databases. Others used national legal archives or court documents. Few used retrospective clinical logs from academic hospitals. This lack of homogeneity created a barrier for consistent statistical evaluation and aggregation.

Across all studies, 20,498 legal cases and 10,567 claims, on average, surgeons were successful in 53% to 56% of lawsuits. The CIs varied between 38% and 69%, depending on study size and region. In some studies, the authors reported very high defendant success rates (eg, Ong et al >60%), whereas others, like Gibstein et al, showed lower values (<40%).^56,58^ This variation reflected differences in legal systems, procedural types, documentation standards, and patient expectations across jurisdictions.^18,64,75,79,80^ Successful defense was more common when surgeons maintained meticulous operative notes, detailed consent forms, and preoperative and postoperative photographs.^72,75^ Ong et al emphasized the impact of expert documentation on court decisions.^58^ Conversely, vague or incomplete records were correlated with legal losses.^72,75^

Jurisdictions with higher litigation culture, such as the United States of America and Canada, demonstrated more frequent claims, greater variance in outcomes, and higher compensation awards. Most of the included studies were from North America. Data from Europe, Asia, and South America were sparse, which limited global generalizability.

The presence and quality of expert witnesses were highlighted in multiple studies as a critical element.^55,59^ In many cases, expert evaluations swayed the decision more than operative details themselves. Litigation outcomes in facial plastic surgery tended to be split, with a mild lean toward the defense when conditions (documentation, procedure planning, and expert support) were adequate.

Facial plastic surgery appears moderately exposed, with outcomes highly dependent on subjective judgments and patient expectations. Facial procedures are uniquely prone to dissatisfaction because of their social and aesthetic prominence, making them frequent targets for litigation. The most common reasons for litigation were poor aesthetic outcomes and intraoperative complications.^81-83^ These aligned with previous reports on surgical liability in aesthetic medicine.^18,19,24,67,80,84^ This underscored the importance of setting realistic patient expectations preoperatively and ensuring comprehensive documentation of informed consent.

Several studies highlighted those cases where informed consent was inadequate had higher settlement rates.^51,58,72,85^ This suggests that, beyond technical surgical performance, clear preoperative discussions about risks and expected outcomes are essential in reducing legal liability.

The findings also emphasized the role of patient selection—some studies noted that litigation-prone often had preexisting psychological concerns, such as body dysmorphic disorder (BDD).^66,86-88^ Implementing screening protocols for such patients could be a preventive strategy.^88-90^

Another important dimension influencing medicolegal outcomes is the quality of the patient–physician relationship. Several studies suggest that beyond technical competence, interpersonal communication and trust play a critical role in patient satisfaction and litigation risk.^29,76^ Effective communication—characterized by active listening, empathy, and transparent discussion of risks and expectations—has been shown to reduce the likelihood of legal claims, even in cases with suboptimal clinical outcomes.^32^ Poor rapport, on the other hand, often escalates patient frustration, particularly when complications arise, or aesthetic results fall short of expectations.^80^ Because facial plastic surgery is highly subjective in outcome, establishing a strong therapeutic alliance through consistent, empathetic dialogue is essential. Incorporating communication training into surgical education and using patient-reported experience measures may further enhance trust and mitigate disputes.^52^

Given the trends observed, several risk-reduction strategies can be considered. Enhanced preoperative counseling is essential. Surgeons should invest time in detailed risk-benefit discussions to manage patient expectations. Maintaining comprehensive documentation, including thorough medical records, informed consent discussions, surgical plans, and postoperative instructions, can provide legal protection. The implementation of objective outcome measures, such as validated aesthetic assessment tools, can help reduce subjective disputes in legal claims. Improved patient selection, particularly through screening for psychological conditions like unrealistic expectations or preexisting BDD, can minimize the likelihood of postsurgical dissatisfaction and litigation. Furthermore, integrating medicolegal education into plastic surgery residency programs could enhance risk management skills among future surgeons.

The practical recommendations presented in this review—enhancing informed consent, improving patient selection, and screening for psychological risk factors—are strongly supported by existing literature. Effective informed consent is not only a legal obligation but also a vital tool for establishing patient trust and managing expectations. Jewell emphasized that a comprehensive, patient-centered consent process that explains risks, alternatives, and expected outcomes significantly reduces the likelihood of litigation, especially when patients are vulnerable to misinterpreting risk information.^32^ Furthermore, Nejadsarvari and Ebrahimi underscored that most legal claims in aesthetic surgery stem from inadequate communication rather than technical errors, advocating for standardized, well-documented consent protocols and legal training for aesthetic surgeons.^31^ Psychological screening, particularly for conditions like BDD, is equally crucial. Pititto et al demonstrated that untreated psychological vulnerabilities in aesthetic surgery patients increase dissatisfaction and legal complaints, suggesting that preoperative evaluations by mental health professionals should be routine practice.^23^ Together, these strategies form an evidence-based framework for reducing medicolegal risk and improving patient safety in facial aesthetic surgery.

Key risk factors contributing to litigation included deficient clinical assessment, failure to recognize symptoms, incorrect diagnosis or treatment, lack of follow-up care, and technical errors.

The findings of this review highlighted the growing medicolegal burden in aesthetic plastic surgery. These results reinforced the need for improved risk management strategies, including enhanced informed consent processes, postoperative follow-up, and legal protections for surgeons practicing in high-volume plastic surgery markets.

### Future Directions

Future research should focus on developing standardized informed consent protocols tailored for facial aesthetic procedures, incorporating digital tools and visual aids. Additionally, creating surgeon training modules on medicolegal risk mitigation, psychological screening tools for high-risk patients, and artificial intelligence–assisted outcome prediction models can improve patient selection and communication. Multinational databases could enhance comparative studies, guiding global best practices in legal risk reduction and patient-centered care in aesthetic surgery.

## CONCLUSIONS

The systematic review underscores the medicolegal risks associated with facial plastic surgery. The findings suggest that surgeons can reduce their legal vulnerability through improved patient communication, meticulous documentation, and appropriate patient selection. Although most malpractice cases result in dismissal, understanding common litigation themes can help shape best practices in surgical care and risk management. The current systematic review provides valuable insights into medicolegal trends in facial plastic surgery, highlighting key risk factors and strategies for litigation prevention.

## Supplemental Material

This article contains supplemental material located online at https://doi.org/10.1093/asj/sjaf082.

## Supplementary Material

sjaf082_Supplementary_Data
