# Supplementary material for: The Medicolegal Challenges of Facial Plastic Surgery: A Systematic Review
Source: Aesthet Surg J. 2025 May 16;45(9):973–84. doi: 10.1093/asj/sjaf082 (PMC12358214; doi:10.1093/asj/sjaf082)
Supplement: sjaf082_Supplementary_Data [file sjaf082_supplementary_data.docx]

*Supplementary Material Appendix 1.*

**THE MEDICOLEGAL CHALLENGES OF FACIAL PLASTIC SURGERY:**

**А SYSTEMATIC REVIEW**

**PICOS framework:**

**•** *Population (P):* Individuals who underwent facial aesthetic surgical procedures.

**•** *Intervention (I):* Evaluation of postoperative complications and medico-legal disputes.

**•** *Comparison (C):* Not applicable

**•** *Outcome (O):* Identification of causes of medico-legal challenges, common complications, and proposed solutions.

**Research Question:**

In patients dissatisfied with facial surgery outcomes (P), how do medicolegal evaluations (I) impact risk management in medical practice (O)?

**Supplemental Table 1**. Eligibility Criteria

| **Inclusion Criteria** | **Exclusion Criteria** |
| --- | --- |
| 1. Adults aged 18 and above, men, and women, underwent facial plastic surgery | 1. Adults, men, and women underwent plastic surgery in other anatomical regions and aesthetic non-surgical procedures |
| 1. Facial plastic or aesthetic surgical procedures (brow lift, upper lid blepharoplasty, lower lid blepharoplasty facelift, rhytidectomy, neck lift, platysmaplasty, rhinoplasty, volume augmentation, genioplasty, malarplasty, lip lift, dimple creation, buccal fat removal) | 1. Aesthetic non-surgical procedures (injectable procedures - injection of hyaluronic acids, botox, hydroxyapatites, laser ablations, tattoo) |
| 1. Complications after facial plastic surgery, unsatisfactory cases categorized under medical malpractice, professional misconduct, litigation for medical negligence, license revocation, claims for moral damages, forensic medical disputes, compensation claims for medical harm, and adverse outcome liability. | 1. Complications due to dental practice and reconstructive surgeries in maxillofacial regions after trauma or congenital and acquired defects and deformities. |
| 1. English articles | 1. Individuals below 18 years |
| 1. 2020 and upwards | 1. Non-humans, In-vitro studies |
| 1. Cases, case series, cohort studies, and descriptive studies | 1. Non-English |
|  | 1. Articles published before 2020 |
|  | 1. Commentary, editorial, comment, review (systematic or otherwise), report, guidelines, position paper, dissertations/theses, trials |

**Supplemental Table 2.** Search Strategy

| **PICO(S) Database**  **Search Strategy** | *Search 1 (P):* Human*  *Search 2 (I):* Face/su [Surgery] OR Surgery, Plastic OR “Plastic Surgery Procedures” OR Esthetics  *Search 3 (C):*  *Search 4 (O):* Liability, Legal OR Malpractice OR Legislation OR Forensic Medicine OR Compensation and Redress  ***Combined Searches***  Search 1 AND Search 2 AND Search 3 AND Search 4 |
| --- | --- |
| **Databases searched** | MEDLINE *(Ovid)*  EMBASE *(Ovid)*  Cochrane Library, including the Central Register of Controlled Trials *(CENTRAL)*  PubMed  Web of Science *(Clarivate Analytics)*  SCOPUS (Elsevier)  Google Scholar *(Advanced search)* |
| ***Grey Literature*** | |
| **Registers** | ClinicalTrials.gov  WHO ICTRP |
| **Websites** | WorldCat  ProQuest Dissertations & Theses (PQDT)  Open Access Theses and Dissertations (OATD)  King's College London Research Portal  SSRN  F1000 |
| **Conference Proceedings** | SCOPUS  Web of Science |
| **Citation searching /**  **Hand-searching** | Web of Science *(Core Collection)*  SCOPUS *(Elsevier)*  Google Scholar  Semantic Scholar  ResearchGate  Citation Chaser |
| **Part of the journals searched** | Keywords in the abstract and title  MeSH terms  Subject Heading Searching *(Medline, Embase)*  Subheadings *(Medline, Embase )* |
| **Years of search** | 2020 onwards  Searches were updated to March 31, 2025 |
| **Language** | English language only |

**Supplemental Table 3.** Search Strategy adapted to each Database

| **Electronic Database** | **Search Strategy Used** | **Filter/**  **Results** |
| --- | --- | --- |
| **MEDLINE**  **via OVID** | Ovid MEDLINE(R) ALL <1946 to February 11, 2025>  1 exp Humans/ 22530037  2 Face/su [Surgery] 5848  3 exp Plastic Surgery Procedures/ 269276  4 exp Surgery, Plastic/ 28848  5 exp Liability, Legal/ 16141  6 exp Malpractice/ 32733  7 exp Legislation/ 1641  8 exp Forensic Medicine/ 109013  9 (Compensation and Redress).mp. [mp=title, book title, abstract, original title, name of substance word, subject heading word, floating sub-heading word, keyword heading word, organism supplementary concept word, protocol supplementary concept word, rare disease supplementary concept word, unique identifier, synonyms, population supplementary concept word, anatomy supplementary concept word] 3236  10 Esthetics/ 15419  11 "Cosmetic surger*".mp. [mp=title, book title, abstract, original title, name of substance word, subject heading word, floating sub-heading word, keyword heading word, organism supplementary concept word, protocol supplementary concept word, rare disease supplementary concept word, unique identifier, synonyms, population supplementary concept word, anatomy supplementary concept word] 2951  12 2 or 3 or 4 or 10 or 11 300127  13 5 or 6 or 7 or 8 or 9 155273  14 1 and 12 and 13 546  15 14 and 2020:2025.(sa_year)  *Searches were updated to March 31, 2025* | **Language:** no filter  **Publication date:** 2020  onwards  **Hits: 56** |
| **Embase**  **via OVID** | Embase <1974 to 2025 February 11>  1 face/su [Surgery] 1298  2 exp plastic surgery/ 341771  3 Plastic Surgery Procedures.mp. 610  4 Cosmetic surger*.mp. 3460  5 exp esthetics/ 24116  6 exp legal liability/ 17041  7 exp malpractice/ 34035  8 Legislation.mp. 89857  9 exp forensic medicine/ 58735  10 (Compensation and Redress).mp. [mp=title, abstract, heading word, drug trade name, original title, device manufacturer, drug manufacturer, device trade name, keyword heading word, floating subheading word, candidate term word] 137  11 3 or 4 or 5 27860  12 6 or 7 or 8 or 9 or 10 190789  13 11 and 12 158  14 13 and 2020:2025.(sa_year).  *Searches were updated to March 31, 2025* | **Language:** no filter  **Publication date:** 2020 onwards  **Hits:37** |
| **Cochrane Library, including Central Register of Controlled Trials (CENTRAL)** | (esthetic surgery OR "face surgery" OR facelift OR rhinoplasty OR plastic surgery):ti,ab,kw AND (litigation OR forensic OR legal):ti,ab,kw (Word variations have been searched)  *Searches were updated to March 31, 2025* | **Language:** no filter  **Publication date:** 2020 onwards  **Hits: 17** |
| **PubMed** | ("face surger*" OR "face plastic surger*" OR "cosmetic surger*" OR "*esthetic* surgery*") AND (Malpractice OR "Forensic medicine" OR "Legislation" OR "Compensation" OR "Legal" OR "Liability")  *Searches were updated to March 31, 2025* | **Language:** no filter  **Publication date:** from 2020 onwards  **Hits: 35** |
| **Web of Science**  Core Collection  *(Clarivate Analytics****)*** | Date Run: Feb 14 2025  A&HCI , ESCI , CPCI-SSH , CPCI-S , SCI-EXPANDED , SSCI  **"face surgery" OR "plastic surgery" OR "plastic surgery procedures" (All Fields) and forensic medicine OR malpractice OR legal OR liability OR legislation (All Fields) and complication (All Fields)**  Timespan: 2020-01-01 onwards  *Searches were updated to March 31, 2025* | **Language:** no filter  **Publication date:** from 2020 onwards  **Hits: 28** |
| **Scopus**  **(Elsevier)** | "aesthetic surgery" AND "malpractice" AND "face" AND "plastic surgery" PUBYEAR > 2019 AND PUBYEAR < 2026  *Searches were updated to March 31, 2025* | **Language:** no filter  **Publication date:** from 2020 onwards  **Hits:58** |
| **Google Scholar**  *(Advanced search)* | Date Run: Feb 03 2025  Timespan: 2020-2025  1/ Forensic * OR Legal OR malpractice  2/Plastic Surgery*  3/face*  Forensic * or Legal face * Plastic OR * "Face * Surgery*"  *Searches were updated to March 31, 2025* | **Language:** no filter  **Publication date:** from 2020 onwards  **Hits: 41** |
| **Citation searching** | The literature search was supplemented by examining references in the relevant articles found. We used the Science Citation Index and Social Sciences Citation Index in Web of Science to track relevant citations.  *Searches were updated to March 31, 2025* | **Language:** no filter  **Publication date:** from 2020 onwards  **Hits: 12** |
| ***Grey literature*** | | |
| **ClinicalTrials.gov** | <https://clinicaltrials.gov/>  Searched 3 February 2025 (1 records) | No filter |
| **WHO ICTRP** | <https://trialsearch.who.int/>  Searched February 2025 (1 records) | No filter |
| **WorldCat** | <https://search.worldcat.org/topics/welcome>  Searched February 2025 (1 records)  "Face plastic surgery"  Searches 31 March 2025 | No filter |

**Supplemental Table 4.** PICO(S) Search Strategy

| **Concept 1:** | **Concept 2:** | **Concept 3:** |
| --- | --- | --- |
| humans | facе surgery  OR  plastic surgery  OR  plastic surgery procedures  OR  cosmetic surgery  OR  esthetics | forensic medicine  OR  malpractice  OR  legislation  OR  liability, legal  OR  compensation and redress |

**Supplemental Table 5.** MeSH Terms

| **MeSH Component** | **MeSH Terms** |
| --- | --- |
| MeSH 1 | Human |
| MeSH 2 | Plastic Surgery Procedures  Surgery,  Plastic  Esthetics |
| MeSH 3 | Liability, Legal  Malpractice  Legislation  Forensic medicine |

**Search strategy (MeSH Terms)**

(human*) AND ((Face/su) OR (Surgery, Plastic[MeSH Terms]) OR (Plastic Surgery Procedures[MeSH Terms]) OR ("Cosmetic surger*") OR (Esthetics[MeSH Terms]) AND ((Liability, Legal[MeSH Terms]) OR (Malpractice[MeSH Terms]) OR (Legislation[MeSH Terms]) OR (Forensic Medicine[MeSH Terms]) OR (Compensation and Redress[MeSH Terms]))

**Supplemental Table 6.** Risk of bias assessment of case reports, cohort studies (JBI CRITICAL APPRAISAL CHECKLIST FOR CASE REPORTS)

<https://jbi.global/sites/default/files/2019-05/JBI_Critical_Appraisal-Checklist_for_Case_Reports2017_0.pdf>

| **Study** | | **Criteria** | | | | | | | |
| --- | --- | --- | --- | --- | --- | --- | --- | --- | --- |
|  |  | **1** | **2** | **3** | **4** | **5** | **6** | **7** | **8** |
|  |  | Were the patient’s demographic characteristics clearly described? | Was the patient’s history clearly described and presented as a timeline? | Was the current clinical condition of the patient on presentation clearly described? | Were diagnostic tests or assessment methods and the results clearly described? | Were the intervention(s) or treatment procedure (s) clearly described? | Was the post-intervention clinical condition clearly described? | Were adverse events (harms) or unanticipated events identified and described? | Does the case report provide takeaway lessons? |
| Ven  ditto et al.^60^ | *Case 1* | Yes | Yes | Yes | Yes | Yes | Yes | Yes | Yes |
|  | *Case 2* | Yes | Yes | Yes | Yes | Yes | Yes | Yes | Yes |
|  | *Case 3* | Yes | Yes | Yes | Yes | Yes | Yes | Yes | Yes |
|  | *Case 4* | Yes | Yes | Yes | Yes | Yes | Yes | Yes | Yes |
|  | *Case 5* | Yes | Yes | Yes | Yes | Yes | Yes | Yes | Yes |
|  | *Case 6* | Yes | Yes | Yes | Yes | Yes | Yes | Yes | Yes |
|  | *Case 7* | Yes | Yes | Yes | Yes | Yes | Yes | Yes | Yes |
|  | *Case 8* | Yes | Yes | Yes | Yes | Yes | Yes | Yes | Yes |
|  | *Case 9* | Yes | Yes | Yes | Yes | Yes | Yes | Yes | Yes |
|  | *Case 10* | Yes | Yes | Yes | Yes | Yes | Yes | Yes | Yes |
| Defraia et al.^63^ | | Yes | Yes | Yes | Yes | Yes | Yes | Yes | Yes |
| Nagano et al.^65^ | | Yes | Yes | Yes | Yes | Yes | Yes | Yes | Yes |
| Dhooghe  et al.^71^ | | Yes | Yes | Yes | Yes | Yes | Yes | Yes | Yes |

*Note:* *Yes /No/ Unclear /Not applicable

**Supplemental Table 7**. Assessment of risk of bias with the ROBINS-I tool

<https://sites.google.com/site/riskofbiastool/welcome/robins-i-v2?authuser=0>


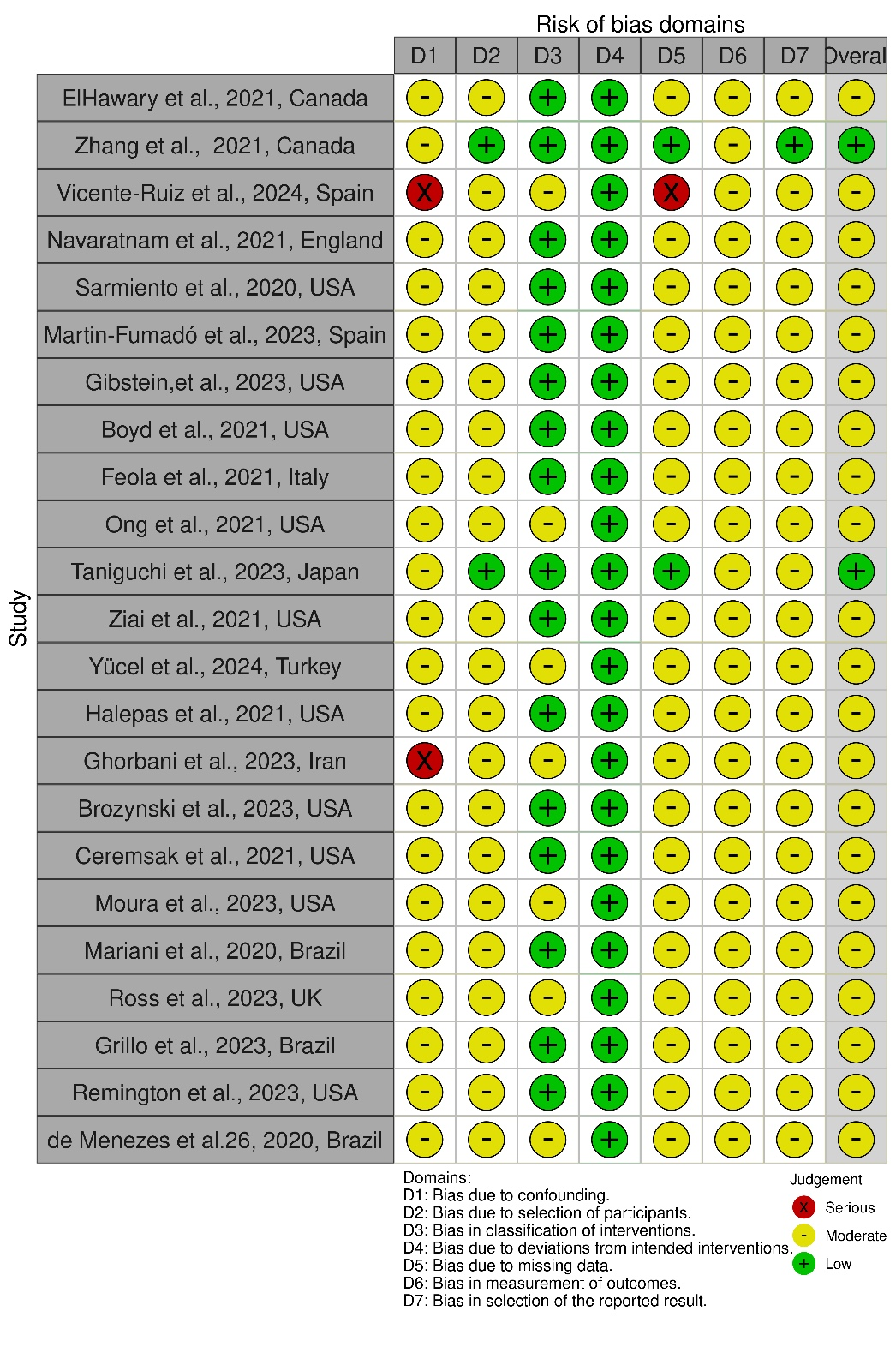


**Supplemental Table 8.** Risk of Bias Assessment Summary

| **№** | **Studies** | **Summary Quality** |
| --- | --- | --- |
|  | El Hawary et al.^46^ 2021, Canada | 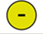 |
|  | Zhang et al.^47^, 2021, Canada | 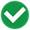 |
|  | Vicente-Ruiz et al.^61^, 2024, Spain | 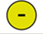 |
|  | Navaratnam et al.^49^, 2021, England | 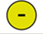 |
|  | Sarmiento et al.^50^, 2020, USA | 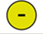 |
|  | Martin-Fumadо et al.^62^, 2023, Spain | 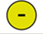 |
|  | Gibstein et al.^51^, 2023, USA | 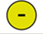 |
|  | Boyd et al.^52^, 2021, USA | 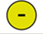 |
|  | Feola et al.^48^, 2021, Italy | 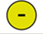 |
|  | Ong et al.^53^, 2021, USA | 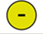 |
|  | Taniguchi et al.^64^, 2023, Japan | 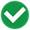 |
|  | Ziai et al.^54^, 2021, USA | 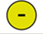 |
|  | Yücel et al.^69^, 2024, Turkey | 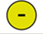 |
|  | Halepas et al.^55^, 2021, USA | 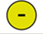 |
|  | Ghorbani et al.^72^, 2023, Iran | 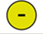 |
|  | Brozynski et al.^56^, 2023, USA | 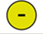 |
|  | Ceremsak et al.^57^, 2021, USA | 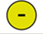 |
|  | Moura et al.^58^, 2023, USA | 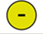 |
|  | Mariani et al.^66^, 2020, Brazil | 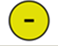 |
|  | Ross et al.^70^, 2023, UK | 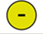 |
|  | Grillo et al.^67^, 2023, Brazil | 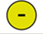 |
|  | Remington et al.^59^, 2023, USА | 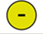 |
|  | Nagano et al.^65^, 2024, Japan | 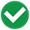 |
|  | Venditto et al.^60^, 2024, USA | 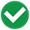 |
|  | Defraia et al.^63^, 2024, Italy | 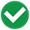 |
|  | Dhooghe et al.^71^, 2023, Belgium | 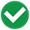 |
|  | de Menezes et al.^68^, 2020, Brazil | 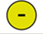 |

*Note:* Review authors’ judgements about each risk of bias item for each included study:*
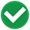
- Low;
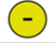
- Moderate;
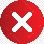
 - High;
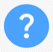
- Unclear.

**Supplemental Table 9**. Included Studies

| **№** | **Study** |
| --- | --- |
|  | ElHawary H, Aldien AS, Gorgy A, Salimi A, Gilardino MS. Dissecting Medical Litigation: An Analysis of Canadian Legal Cases in Plastic Surgery. Plast Surg (Oakv). 2023;31(2):161-167. Doi: 10.1177/22925503211034835. |
|  | Zhang Z, Calder L, Finestone PJ, Liu R, Bucevska M, Arneja JS. Medico-legal Closed Case Trends in Canadian Plastic Surgery: A Retrospective Descriptive Study. Plast Reconstr Surg Glob Open. 2021;9(8):e3754. Doi: 10.1097/GOX.0000000000003754. |
|  | Vicente-Ruiz M, Hontanilla B. A Novel Method for Identifying Patients with High Risk of Litigation in Plastic Surgery: Introducing the FATIMA Acronym. Aesthetic Plast Surg. 2025;49(1):400-406. Doi: 10.1007/s00266-024-04476-2. |
|  | Navaratnam AV, Pendolino AL, Kaura A, Nijim J, Machin JT, Briggs TWR, Marshall A, Randhawa PS, Andrews PJ. Lessons from rhinology and facial plastic surgery clinical negligence claims in England 2013-2018. J Laryngol Otol. 2022;136(12):1177-1182. Doi: 10.1017/s0022215121003959. |
|  | Sarmiento S, Wen C, Cheah MA, Lee S, Rosson GD. Malpractice Litigation in Plastic Surgery: Can We Identify Patterns? Aesthet Surg J. 2020;40(6): NP394-NP401. Doi: 10.1093/asj/sjz258. |
|  | Martin-Fumadó C, Benet-Travé J, Güerri-Fernández R, López-Ojeda A, Bermejo-Segú O, Arimany-Manso J. Professional Liability in Plastic Surgery: A Change of Scenario in Medical Professional Liability in Spain. Plast Reconstr Surg Glob Open. 2023;11(6):e5054. Doi: 10.1097/GOX.0000000000005054. |
|  | Gibstein AR, Jabori SK, Watane A, Slavin BR, Elabd R, Singh D. Do Plastic Surgery Residents Get Sued? An Analysis of Malpractice Lawsuits. Plast Reconstr Surg Glob Open. 2023;11(1):e4721. Doi: 10.1097/GOX.0000000000004721. |
|  | Boyd JB, Moon HK, Martin S, Mastrogiovanni DB. Plastic Surgery and the Malpractice Industry. Plast Reconstr Surg. 2021;147(1):239-247. Doi: 10.1097/PRS.0000000000007497. |
|  | Feola A, Minotti C, Marchetti D, Caricato M, Capolupo GT, Marsella LT, La Monaca G. A Five-Year Survey for Plastic Surgery Malpractice Claims in Rome, Italy. Medicina (Kaunas). 2021;57(6):571. Doi: 10.3390/medicina57060571. |
|  | Ong AA, Kelly A, Castillo GA, Carr MM, Sherris DA. Characterization of Medical Malpractice Litigation After Rhinoplasty in the United States. Aesthet Surg J. 2021;41(10):1132-1138. Doi: 10.1093/asj/sjaa380. |
|  | Taniguchi K, Watari T, Nagoshi K. Characteristics and trends of medical malpractice claims in Japan between 2006 and 2021. PLOS ONE. 2023;18(12): e0296155. Doi:10.1371/journal.pone.0296155. |
|  | Ziai K, Patel S, Thomas S, Shokri T, Lighthall JG. A Century in Review: Medicolegal Implications of Facial Nerve Paralysis. Facial Plast Surg Aesthet Med. 2021;23(6):417-421. Doi: 10.1089/fpsam.2020.0390. |
|  | Yücel, Ahmet Faruk1; Cenger, Cüneyt Destan2; Arıncı, Rifat Atilla3; Tüzün, Birgül4. Evaluation of Medical Malpractice Claim Reports Regarding Aesthetic Medical Intervention Cases. Turkish Journal of Plastic Surgery. 2024;32(2):49-53. Doi: 10.4103/tjps.tjps_59_23. |
|  | Halepas S, Muchemi F, Higham ZL, Ferneini EM. The Past Decade in Courts, What Oral-Maxillofacial Surgery Should Know About Facial Cosmetic Surgery. J Oral Maxillofac Surg. 2021;79(8):1743-1749. Doi: 10.1016/j.joms.2021.04.007. |
|  | Ghorbani F, Ahmadi H, Davar G. Patient dissatisfaction following rhinoplasty: a 10-year experience in Iran. Maxillofac Plast Reconstr Surg. 2023;45(1):7. Doi: 10.1186/s40902-022-00369-z. |
|  | Brozynski M, Di Via Loschpe A, Oleru O, Seyidova N, Rew C, Taub PJ. Never events in plastic surgery: An analysis of surgical burns and medical malpractice litigation. Burns. 2024;50(5):1232-1240. Doi: 10.1016/j.burns.2024.02.007. |
|  | Ceremsak J, Miller LE, Gomez ED. A Review of Otolaryngology Malpractice Cases with Associated Court Proceedings from 2010 to 2019. Laryngoscope. 2021;131(4):E1081-E1085. Doi: 10.1002/lary.29232. |
|  | Moura, Steven P. MA; Shaffrey, Ellen C. MD; Lam, Chloe S. BS; Wirth, Peter J. MD; Attaluri, Pradeep K. MD; Rao, Venkat K. MD, MBA. Out-of-scope Cosmetic Surgery: A Review of Malpractice Lawsuits against Nonplastic Surgeons. Plastic & Reconstructive Surgery-Global Open. 2023;11(3):e4873. Doi: 10.1097/GOX.0000000000004873. |
|  | Mariani PC, Constantino CF, Nunes R. Classification of plastic surgery malpractice complaints brought before the São Paulo Medical Board that were treated as professional-misconduct cases: a cross-sectional study. Sao Paulo Med J. 2020;138(2):140-145. Doi: 10.1590/1516-3180.2019.0363.09122019. |
|  | Ross T, Arwyn-Jones J, Navaratnam AV, Pendolino AL, Randhawa PS, Andrews P, Saleh HA. Litigation in Septorhinoplasty Surgery: A Pan-Specialty Review of National Health Service (the United Kingdom) Data. Facial Plast Surg. 2023;39(2):142-147. Doi: 10.1055/a-1910-0604. |
|  | Grillo R, Brozoski MA, Naclério-Homem MDG. The importance of written informed consent in facial cosmetic surgery litigation. J Craniomaxillofac Surg. 2023;51(7-8):403-406. Doi: 10.1016/j.jcms.2023.08.007. |
|  | Remington AC, Schaffer A, Hespe GE, Yugar CJ, Sherif R, Vercler CJ. Understanding Factors Associated with Paid Malpractice Claims in Plastic Surgery. Plast Reconstr Surg. 2024;153(3):644e-649e. Doi: 10.1097/PRS.0000000000010593. |
|  | Nagano S, Unuma K, Makino Y, Mori H, Uemura K. Acute upper airway obstruction due to cervical hematoma after cervicofacial liposuction. J Forensic Leg Med. 2024;104:102697. Doi: 10.1016/j.jflm.2024.102697. |
|  | Venditto CM, Grotting JC, Auersvald A, Johnson DJ, Labbé D, Hodgkinson D, Barrera A, Warren RJ, Botti G, Von Laeken N, Bald M. Complete Hemifacial Paralysis Post-facelift: Making Sense of a Plastic Surgeon's Worst Nightmare. Aesthet Surg J. 2024 15;44(3):256-264. Doi: 10.1093/asj/sjad337. |
|  | Defraia B, Focardi M, Grassi S, Chiavacci G, Faccioli S, Romano GF, Bianchi I, Pinchi V, Innocenti A. Negative Outcomes of Blepharoplasty and Thyroid Disorders: Is Compensation Always Due? A Case Report with a Literature Review. Diseases. 2024;12(4):75. Doi: 10.3390/diseases12040075. |
|  | Dhooghe NS, Maes S, Depypere B, Claes KEY, Coopman R, Kubat B, Piette MH, Monstrey S. Fat Embolism After Autologous Facial Fat Grafting. Aesthet Surg J. 2022;42(3):231-238. Doi: 10.1093/asj/sjab252. |
|  | de Menezes, Jorge Antônio et al. Legal disputes involving plastic surgeries in Brazil: Main causes of patients ‘complaints, trial outcomes, and influence of the expert report on the results of appellate courts. Journal of Plastic, Reconstructive & Aesthetic Surgery. 2020;73(6):1174 -1205. Doi:10.1016/j.bjps.2020.01.035. |

**Supplemental Table 10.** Excluded Studies and Reasons

| **№** | **Study** | **Reason** |
| --- | --- | --- |
|  | Borzabadi-Farahani A, Mosahebi A, Zargaran D. A Scoping Review of Hyaluronidase Use in Managing the Complications of Aesthetic Interventions. Aesthetic Plast Surg. 2024;48(6):1193-1209. Doi: 10.1007/s00266-022-03207-9. | *Review* |
|  | Kyriazidis I, Spyropoulou GA, Zambacos G, Tagka A, Rakhorst HA, Gasteratos K, Berner JE, Mandrekas A. Adverse Events Associated with Hyaluronic Acid Filler Injection for Non-surgical Facial Aesthetics: A Systematic Review of High Level of Evidence Studies. Aesthetic Plast Surg. 2024;48(4):719-741. Doi: 10.1007/s00266-023-03465-1. | *Review* |
|  | Davison SP, Evans G, Ball E, Newman A, Sotile W. Control versus Choice in Deciding Career Pathway in Plastic Surgery: The Perfect Ratio. Plast Reconstr Surg Glob Open. 2024;12(10):e6240. Doi:10.1097/GOX.0000000000006240 | *Review* |
|  | Chappell AG, Kane RL, Wood SM, Wescott AB, Chung KC. Representation of Ethics in the Plastic Surgery Literature: A Systematic Review. Plast Reconstr Surg. 2021;148(2):289e-298e. Doi:10.1097/PRS.0000000000008232 | *Review* |
|  | Pinkiewicz M, Dorobisz K, Zatoński T. A Comprehensive Approach to Facial Reanimation: A Systematic Review. J Clin Med. 2022;11(10):2890. Doi:10.3390/jcm11102890 | *Review* |
|  | Sharif-Askary B, Carlson AR, Van Noord MG, Marcus JR. Incidence of Postoperative Adverse Events after Rhinoplasty: A Systematic Review. Plast Reconstr Surg.2020;145(3):669-684. Doi:10.1097/PRS.0000000000006561 | *Review* |
|  | Beauvais D, Ferneini EM. Complications and Litigation Associated With Injectable Facial Fillers: A Cross-Sectional Study. J Oral Maxillofac Surg. 2020;78(1):133-140.Doi: 10.1016/j.joms.2019.08.003. | The study is not related to aesthetic surgery.  *(Unspecific)* |
|  | Halepas S, Lee KC, Higham ZL, Ferneini EM. A 20-Year Analysis of Adverse Events and Litigation With Light-Based Skin Resurfacing Procedures. J Oral Maxillofac Surg. 2020;78(4):619-628. Doi: 10.1016/j.joms.2019.12.012. | The study is not related to aesthetic surgery.  *(Unspecific)* |
|  | Zhu D, Wong A, Tham T, Kraus D. The paralyzing legal costs of facial nerve injury in head and neck tumors. Am J Otolaryngol. 2020;41(6):102693. Doi: 10.1016/j.amjoto.2020.102693. | The study is not related to aesthetic surgery.  *(Unspecific)* |
|  | Shah SN, Ramirez AV, Haroun KB, Chaaban MR. Medical Liability in Sinus Surgery: A Westlaw Database Analysis From 2000 to 2017. Am J Rhinol Allergy. 2020;34(5):642-649. Doi: 10.1177/1945892420920479. | The study is not related to aesthetic surgery.  *(Unspecific)* |
|  | Reese AD, Rosi-Schumacher M, Carr MM. Characterization of litigation after tonsillectomy in the United States. Am J Otolaryngol. 2022;43(5):103566. Doi: 10.1016/j.amjoto.2022.103566. | The study is not related to aesthetic surgery.  *(Unspecific)* |
|  | Hyman DA, Lerner J, Magid DJ, Black B. Association of Past and Future Paid Medical Malpractice Claims. JAMA Health Forum. 2023;4(2):e225436. Doi: 10.1001/jamahealthforum.2022.5436. | The study is not related to aesthetic surgery.  *(Unspecific)* |
|  | Luotamo I, Snäll J, Toivari M. Complications and complaints in craniofacial fractures - Finnish national data for 20 years. Acta Odontol Scand. 2024;83:334-339. Doi: 10.2340/aos.v83.40570. | The study is not related to aesthetic surgery.  *(Unspecific)* |
|  | Salimi M, Heidari MB, Ravandi Z, Mosalamiaghili S, Mirghaderi P, Jafari Kafiabadi M, Biglari F, Salimi A, Sabaghzadeh Irani A, Khabiri SS. Investigation of litigation in trauma orthopaedic surgery. World J Clin Cases. 2023;11(5):1000-1008. Doi: 10.12998/wjcc.v11.i5.1000. | The study is not related to aesthetic surgery.  *(Unspecific)* |
|  | Rahman E, Philipp-Dormston WG, Webb WR, Rao P, Sayed K, Sharif AQMO, Yu N, Ioannidis S, Tam E, Rahman Z, Mosahebi A, Goodman GJ. "Filler-Associated Acute Stroke Syndrome": Classification, Predictive Modelling of Hyaluronidase Efficacy, and Updated Case Review on Neurological and Visual Complications. Aesthetic Plast Surg. 2024;48(17):3222-3253. Doi: 10.1007/s00266-024-04202-y. | The study is not related to aesthetic surgery.  *(Unspecific)* |
|  | Crosby ET, Duggan LV, Finestone PJ, Liu R, De Gorter R, Calder LA. Anesthesiology airway-related medicolegal cases from the Canadian Medical Protection Association. Can J Anaesth. 2021;68(2):183-195. Doi: 10.1007/s12630-020-01846-7. | The study is not related to aesthetic surgery.  *(Unspecific)* |
|  | Brozynski M, Seyidova N, Oleru O, Rew C, Roy N, Taub PJ. An Analysis of Medical Malpractice Litigation Involving Mandibular Fractures. FACE. 2023;4(3):386-396. Doi:10.1177/27325016231186303 | The study is not related to aesthetic surgery.  *(Unspecific)* |
|  | Rosi-Schumacher MR, O'Donnell JD, Ong AA, Sherris DA. Litigation Following Surgery for Acute Traumatic Facial Injuries. Cureus. 2024;16(10):e72660. Doi: 10.7759/cureus.72660. | The study is not related to aesthetic surgery.  *(Unspecific)* |
|  | Stratman S, Dover JS, Labadie JG. Cross-sectional analysis of state and federal malpractice and medical liability claims for cutaneous energy-based device procedures, 1985 to 2023. J Am Acad Dermatol. 2024;91(4):742-744. Doi: 10.1016/j.jaad.2024.06.033. | The study is not related to aesthetic surgery.  *(Unspecific)* |
|  | Kim MJ, Shin SH, Park JY. Medicolegal implications from litigations involving necrotizing fasciitis. Ann Surg Treat Res. 2020;99(3):131-137. Doi: 10.4174/astr.2020.99.3.131. | The study is not related to aesthetic surgery.  *(Unspecific)* |
|  | Kalambe Ghate S, Kalambe A, Maldhure S. Auricular haematoma an avoidable cosmetic deformity: A chance or negligence. Am J Otolaryngol. 2022;43(1):103232. Doi: 10.1016/j.amjoto.2021.103232. | The study is not related to aesthetic surgery.  *(Unspecific)* |
|  | Mamoon OKA, Asaad MK, Hussein ASAl. Topical Application of Povidone Iodine to Minimize Post-Appendectomy Wound Infection. Indian Journal of Forensic Medicine &amp; Toxicology.2021;15(4):1743-1747.  Doi: 10.37506/ijfmt.v15i4.16956. | The study is not related to aesthetic surgery.  *(Unspecific)* |
|  | Gusti AKRH, Pujiyono, Siska DS. Forensic and Toxicological Tests for Protecting the Patients of Esthetic Beauty Clinic as the Constitutional Right in Indonesia. Indian Journal of Forensic Medicine &amp; Toxicology. 2020;14(2):2543-2547. Doi:10.37506/ijfmt.v14i2.3498. | The study is not related to aesthetic surgery.  *(Unspecific)* |
|  | James TA, Fan B. ASO Author Reflections: National Analysis of Breast Surgery Malpractice Cases: A Teachable Moment? Ann Surg Oncol. 2021:28, 8116-8117. Doi:10.1245/s10434-021-10362-x | The study is related to plastic surgery of the breast.  *(Unspecific)* |
|  | Hery D, Schwarte B, Patel K, Elliott JO, Vasko S. Plastic Surgery Tourism: Complications, Costs, and Unnecessary Spending? Aesthet Surg J Open Forum. 2023;6:ojad113. Doi: 10.1093/asjof/ojad113. | The study is not related to complications of aesthetic surgery.  *(Unspecific)* |
|  | Reese A, Navarro A, Kozlowski K, Singh S, Vu G, Neimanis S, Burke M, Morrison C. An Updated Analysis of Body Contouring Malpractice Cases. Ann Plast Surg. 2024;92(4S Suppl 2):S275-S278. Doi: 10.1097/SAP.0000000000003870. | The study is not related to aesthetic surgery of the face.  *(Unspecific)* |
|  | Neder Netto J, Campos RAC, Fujita RR. Patients' understanding of "informed consent" in plastic surgery. Rev Assoc Med Bras (1992). 2021;67(8):1150-1154. Doi: 10.1590/1806-9282.20210545. | The study is not related to complications of aesthetic surgery.  *(Unspecific)* |
|  | White-Dzuro CG, Glassman GE, Patrinely JR, Walker S, Stratton S, Domenico HJ, Galloway M, Pichert JW, Perdikis G, Cooper WO. Coworker Reports about Unprofessional Behavior in Plastic Surgery. Plast Reconstr Surg. 2023;151(4):901-907.  Doi: 10.1097/PRS.0000000000010016. | The study is not related to aesthetic surgery.  *(Unspecific)* |
|  | Dalmar M, El Sheikh M, Baker R, Uppal R. Managing complications following cosmetic surgery after the COVID pandemic: A study of a year at an NHS plastic surgery unit. J Plast Reconstr Aesthet Surg. 2024;88:47-50. Doi: 10.1016/j.bjps.2023.10.135. | The study is not related to complications of facial aesthetic surgery.  *(Unspecific)* |
|  | Ahmed MB, Almohannadi FS, Shraim BA, Aljassem G, Al-Lahham S, Alsherawi A. Surgical Written Consent in Aesthetic Plastic Surgery: A Plastic Center Audit of Surgical Consent Standards. Cureus. 2024;16(1):e51701. Doi: 10.7759/cureus.51701. | The study is related to aesthetic plastic surgery but focuses on obtaining informed consent.  *(Unspecific)* |
|  | Facchin F, Pagani A, Perozzo FAG, Scarpa C, Bassetto F, Vindigni V. Litigation Cases After Post-Bariatric Surgery: Lesson from the Past. Aesthetic Plast Surg. 2023;47(6):2479-2485. Doi: 10.1007/s00266-023-03287-1. | The study focuses on complications associated with Post-Bariatric Surgery.  *(Unspecific)* |
|  | Koziej M, Polak J, Hołda J, Trybus M, Hołda M, Kluza P, Moskała A, Chrapusta A, Walocha J, Woźniak K. The Arteries of the Central Forehead: Implications for Facial Plastic Surgery. Aesthet Surg J. 2020;40(10):1043-1050. Doi: 10.1093/asj/sjz295. | The study is not related to aesthetic surgery.  *(Unspecific)* |
|  | Zhang Z, Hawary HE, Oxley P, Gilardino MS, Arneja JS. What Impact Do Medicolegal Complaints Have on Canadian Plastic Surgeons? Plastic Surgery. 2024;0(0). Doi:10.1177/22925503241300337 | The study is not related to aesthetic surgery.  *(Unspecific)* |
|  | Lim B, Seth I, Marcaccini G, Susini P, Cuomo R, Rozen WM. Regulatory frameworks in plastic and cosmetic surgery: a comparative scoping review across Australia, United Kingdom, and Italy. Gland Surg. 2024;13(8):1561-1572. Doi: 10.21037/gs-24-244. | The study is based on the study Regulatory frameworks in plastic and cosmetic surgery.  *(Unspecific)* |
|  | Strickler AG, Shah P, Bajaj S, Mizuguchi R, Nijhawan RI, Odueyungbo M, Rossi A, Ratner D. Preventing complications in dermatologic surgery: Presurgical concerns. J Am Acad Dermatol. 2021;84(4):883-892. Doi: 10.1016/j.jaad.2020.10.099. | The study focuses on Preventing Complications in Dermatologic Surgery.  *(Unspecific)* |
|  | Arlette JP, Froese AL, Singh JK. Soft Tissue Filler Therapy and Informed Consent: A Canadian Review. J Cutan Med Surg. 2022;26(1):50-56. Doi: 10.1177/12034754211032542. | The study is not related to aesthetic surgery.  *(Unspecific)* |
|  | Deng K, Deng X, Luo H, Chen L, Liu Y, Wang J, Huang M, Hu J, Li T, Zhou J. Academic visualization study of aesthetic medicine management and related legal research since 2000. J Cosmet Dermatol. 2024;23(8):2697-2710. Doi: 10.1111/jocd.16327. | The study is not related to aesthetic surgery.  *(Unspecific)* |
|  | Tseng CC, Patel R, Desai AD, Shah VP, Talmor G, Paskhover B. Assessing Patient Satisfaction Following Blepharoplasty Using Social Media Reviews. Aesthet Surg J. 2022;42(3):NP179-NP185.  Doi: 10.1093/asj/sjab345. | The study is related to the evaluation of plastic surgery results in social networks.  *(Unspecific)* |
|  | Fritz CG, Romeo DJ, Lowery AS, Rajasekaran K. Allegations of Failure to Obtain Informed Consent in Otolaryngology: Evidence-Based Recommendations for Sinus Surgeons. Am J Rhinol Allergy. 2023;37(3):330-336.  Doi: 10.1177/19458924221148566. | The study is related to Otolaryngology and not facial plastic surgery.  *(Unspecific)* |
|  | Shakir S, Kozak GM, Nathan SL, Davis H, Whitely C, Broach RB, Fosnot J. The Role of a Resident Aesthetic Clinic in Addressing the Trainee Autonomy Gap. Aesthet Surg J. 2020;40(5):NP301-NP311. Doi: 10.1093/asj/sjz324. | The study is not related to aesthetic surgery.  *(Unspecific)* |
|  | Brown E, Choi J, Sairi T. Resident Involvement in Plastic Surgery: Divergence of Patient Expectations and Experiences with Surgeon's Attitudes and Actions. J Surg Educ. 2020;77(2):291-299. Doi: 10.1016/j.jsurg.2019.10.008. | The study is not related to aesthetic surgery.  *(Unspecific)* |
|  | Pititto F, Pulin G, Paladini E, Bellacicco R, Marrone M. The Cosmetic Surgery in Adolescents: Psychological, Social, and Medico-Legal Responsibilities, Aesthetic Surgery Journal, 2025; sjaf025. Doi:10.1093/asj/sjaf025. | The study is not related to aesthetic surgery.  *(Unspecific)* |
|  | Budini V, Zanettin C, Brambullo T, Bassetto F, Vindigni V. Aesthetic Surgery Tourism: An Opportunity or a Danger? Aesthetic Plast Surg. 2024;48(19):3914-3920. Doi: 10.1007/s00266-024-04117-8. | The study is not related to aesthetic surgery.  *(Unspecific)* |
|  | Lange MRSS, Silva RF, Ortiz AG, Paranhos LR, Dias PEM, Franco A. Lawsuits after dermal filler injection registered in the state Court of São Paulo. Bioscience Journal. 2023:39;e39069. Doi: 10.14393/BJ-v39n0a2023-67949. | The study is not related to aesthetic surgery.  *(Unspecific)* |
|  | Yıldırım MŞ, Koç Yıldırım S. Insights From the Supreme Court Decisions: Undesirable Consequences After Minimally Invasive Cosmetic Interventions in Türkiye. J Cosmet Dermatol. 2025:24; e16588. Doi:10.1111/jocd.16588 | The study is not related to aesthetic surgery.  *(Unspecific)* |
|  | Marion T, Werbel T, Torres A. Reducing Legal Risks and Social Media Issues for Cosmetic Surgery. Facial Plast Surg Clin North Am. 2023;31(2):333-340. Doi: 10.1016/j.fsc.2023.01.017. | The study is not related to aesthetic surgery.  *(Unspecific)* |
|  | Dilger AE, Sykes JM. Unhappy Patients Can Turn into Angry Patients: How to Deal with Both. Facial Plast Surg Clin North Am. 2020;28(4):461-468. Doi: 10.1016/j.fsc.2020.06.004. | The study is not related to aesthetic surgery.  *(Unspecific)* |
|  | Stephen D Bresnick, Highly Publicized Litigation Against Doctors: How Plastic Surgeons Should Protect Themselves and Their Patients. Aesthetic Surgery Journal. 2023;43(4):NP297-NP299. [Doi:10.1093/asj/sjac324](https://doi.org/10.1093/asj/sjac324) | The study is not related to aesthetic surgery.  *(Unspecific)* |
|  | راسخ جهرمی, اطهر, درودچی, معتضدیان, احمدی, کلانی, ... & قائدی. (2022). بررسی فراوانی شکایات از پزشکان جراح پلاستیک ارجاع شده به سازمان پزشکی قانونی استان فارس بین سال های 1392 تا 1399. مجله علوم پزشکی پارس, 19(4), 19-25.‎ | not English |
|  | Rasekhjahromi A, Doroudch AR, Motazedian G, Ahmadi F, Kalani N, Ghaedi, M. Frequency of Litigations from plastic surgeon physician's refering to Fars provience forensic medicine organization between 2013 and 2020. Pars Journal of Medical Sciences. 2022;19(4):19-25. Doi: 10.22034/pjms.2022.700497 | not English |
|  | Slobogin CS, Birkbeck College. Dickie Orpen and the Visual Culture of World War II Plastic Surgery in Britain. dissertation. Birkbeck, University of London; 2021. https://eprints.bbk.ac.uk/id/eprint/45854 | Unspecific |
